# Gender Differences in the Prevalence and Development of Metabolic Syndrome in Chinese Population with Abdominal Obesity

**DOI:** 10.1371/journal.pone.0078270

**Published:** 2013-10-23

**Authors:** Shaoyong Xu, Bin Gao, Ying Xing, Jie Ming, Junxiang Bao, Qiang Zhang, Yi Wan, Qiuhe Ji, on behalf of the China National Diabetes and Metabolic Disorders Study Group

**Affiliations:** 1 Department of Endocrinology, the First Affiliated Hospital of Fourth Military Medical University, Xi’an, China; 2 Department of Aerospace Physiology, Fourth Military Medical University, Xi’an, China; 3 Department of Orthopedics, Chinese PLA general hospital, Beijing, China; 4 Department of Health Statistics, School of Public Health, Fourth Military Medical University, Xi’an, China; University of Leicester, United Kingdom

## Abstract

**Background:**

Not all the people with metabolic syndrome (MS) have abdominal obesity (AO). The study aimed to investigate gender differences in the prevalence and development of MS in Chinese population with abdominal obesity, which has rarely been reported.

**Methods:**

Data were obtained from the 2007-08 China National Diabetes and Metabolic Disorders Study, and participants were divided into two samples for analysis. Sample 1 consisted of 19,046 people with abdominal obesity, while sample 2 included 2,124 people meeting pre-specified requirements. Survival analysis was used to analyze the development of MS.

**Results:**

The age-standardized prevalence of MS in Chinese population with AO was 49.5%. The prevalence in males (73.7%) was significantly higher than that in females (36.9%). Males had significantly higher proportions of combinations of three or four MS components than females (36.4% *vs*. 30.2% and 18.4% *vs*. 5%, respectively). MS developed quick at first and became slow down later. Half of the participants with AO developed to MS after 3.9 years (95% CI: 3.7–4.1) from the initial metabolic abnormal component, whereas 75% developed to MS after 7.7 years (95% CI: 7.5–7.9).

**Conclusion:**

Compared with females, Chinese males with AO should receive more attention because of their higher prevalence of MS and its components, more complex and risky combinations of abnormal components, and faster development of MS.

## Introduction

Metabolic syndrome (MS) is a cluster of metabolic risk factors including hyperglycemia, hypertension and dyslipidemia [1-4]. In people with MS, the risk of suffering from diabetes and cardiovascular diseases (CVD) is significantly increased [5]. Abdominal obesity (AO) plays an important role in MS [6] and, together with insulin resistance, is considered to be one of the bases of MS [3]. However, not all the people with MS have AO [6]. It is extremely important to study MS in people with AO as the prevalence of obesity increasing [7,8], because, for example, the prevalence of MS in people with AO is significantly higher than that in normal population [9,10]. Furthermore, AO is often accompanied with more complex combinations of abnormal metabolic components. People with AO have a higher risk of suffering from diabetes and cardiovascular diseases [11,12]. China with large populations undergoing rapid transitions to an urbanized and western diet and lifestyle, is particularly susceptible to the public health burden of obesity [13]. AO and MS are rising and attracting more attention [14]. There have been many epidemiological studies of MS in China; however, most of the subjects were normal people or people with diabetes [15-22]. To date, no study focuses on MS among Chinese population with AO.

Previous studies have shown the process from the occurrence of the first component to the development of MS is gradual, and people with AO develop MS faster than people without AO [23]. The risk of having diabetes and cardiovascular diseases (CVD) is significantly enhanced with increased number of abnormal metabolic components [24]. Attention should be paid to people with the appearance of the first abnormal metabolic component, for the subsequent second abnormal metabolic component or the MS arises quickly. For example, physicians are more concerned about how soon people with AO will develop to MS after the appearance of the first abnormal metabolic component. However, there has been no study of the process of development of MS from the first abnormal metabolic component. 

In addition, it was reported that there was gender difference in the prevalence of MS and its components in general population [25,26]. Hence, data obtained from the 2007-08 China National Diabetes and Metabolic Disorders Study were used to investigate gender difference in the prevalence of MS and its components in Chinese population with AO, and to retrospectively explore gender difference in the development process from the first abnormal component to MS.

## Materials and Methods

### Ethics Statement

This study was approved by the institutional review boards from 17 participating institutions, which included First Affiliated Hospital of Fourth Military Medical University, China-Japan Friendship Hospital, Chinese People’s Liberation Army General Hospital, Third Affiliated Hospital of Sun Yat-sen University, People’s Hospital of Peking University, First Affiliated Hospital of Chinese Medical University, People’s Hospital of Shanxi Province, West China Hospital of Sichuan University, Affiliated Sixth People’s Hospital of Shanghai Jiao Tong University, Affiliated Drum Tower Hospital of Medical School of Nanjing University, Xinjiang Uygur Autonomous Region’s Hospital, Fujian Provincial Hospital, Qilu Hospital of Shandong University, Peking University First Hospital, Henan Province People’s Hospital, Second Affiliated Hospital of Harbin Medical University, and Xiangya Second Hospital. Written informed consent was gained from each participant prior to data collection. The 17 institutional review boards’ approvals covered every participant in the study. 

### 2007-08 China National Diabetes and Metabolic Disorders Study

Data were obtained from the 2007-08 China National Diabetes and Metabolic Disorders Study, which was a population-based nationwide and multi-stage stratified sampling investigation performed between July 2007 and June 2008. Detailed information was previously described [27-31]. Briefly, adults aged >20 years with at least 2 years of residency were selected for the study. Signed informed consents were obtained from the participants before the study. After fasting for at least 10 hours at night, participants with no history of diabetes were given a standard 75-g glucose solution, whereas for safety reasons, participants with a self-reported history of diabetes were given a steamed bun that contained approximately 80g of complex carbohydrates [27]. In addition, all participants were subjected to other laboratory tests, such as fasting triglycerides, total cholesterol (TC), and high-density lipoprotein cholesterol (HDL-c) assessments. Professionally trained doctors or nurses measured anthropometric data including height, weight, waist circumference, and blood pressure [27]. A questionnaire was designed to collect demographic data and lifestyle risk factors on all participants. Educational level was categorized as college or above, secondary school, and elementary school or below. Cigarette smoking was defined as having smoked at least 100 cigarettes in one’s lifetime. Alcohol drinking was defined as consuming alcohol at least once per week for a year or more. Physical activity was defined as participating in moderate or vigorous activity for 30 minutes or more per day for at least 3 days a week. Questions in the questionnaire were used to investigate the diagnosis history of abnormal metabolic components, which included “What is your maximum weight? Please specify your age when you reached your maximum weight.” and “Have you ever had diabetes, hypertriglyceridemia, or hypercholesterolemia? If yes, please indicate the date of diagnosis”. 

### Definitions

The definition of MS referred to the International Diabetes Federation standard [3], with AO defined as waist circumference ≥ 90 cm (males) or ≥ 80 cm (females) plus any two of the following four abnormal metabolic components: 1) fasting triglycerides ≥ 1.69 mmol/l or the use of lipid medications; 2) systolic blood pressure ≥ 130 mmHg, diastolic blood pressure ≥ 85 mmHg, or the use of antihypertensive medications; 3) fasting plasma glucose ≥ 5.6 mmol/l or the use of diabetes medications; and 4) HDL-c < 1.04 mmol/l (male) or < 1.29 mmol/l (female).

### Study Population

**Figure 1:**
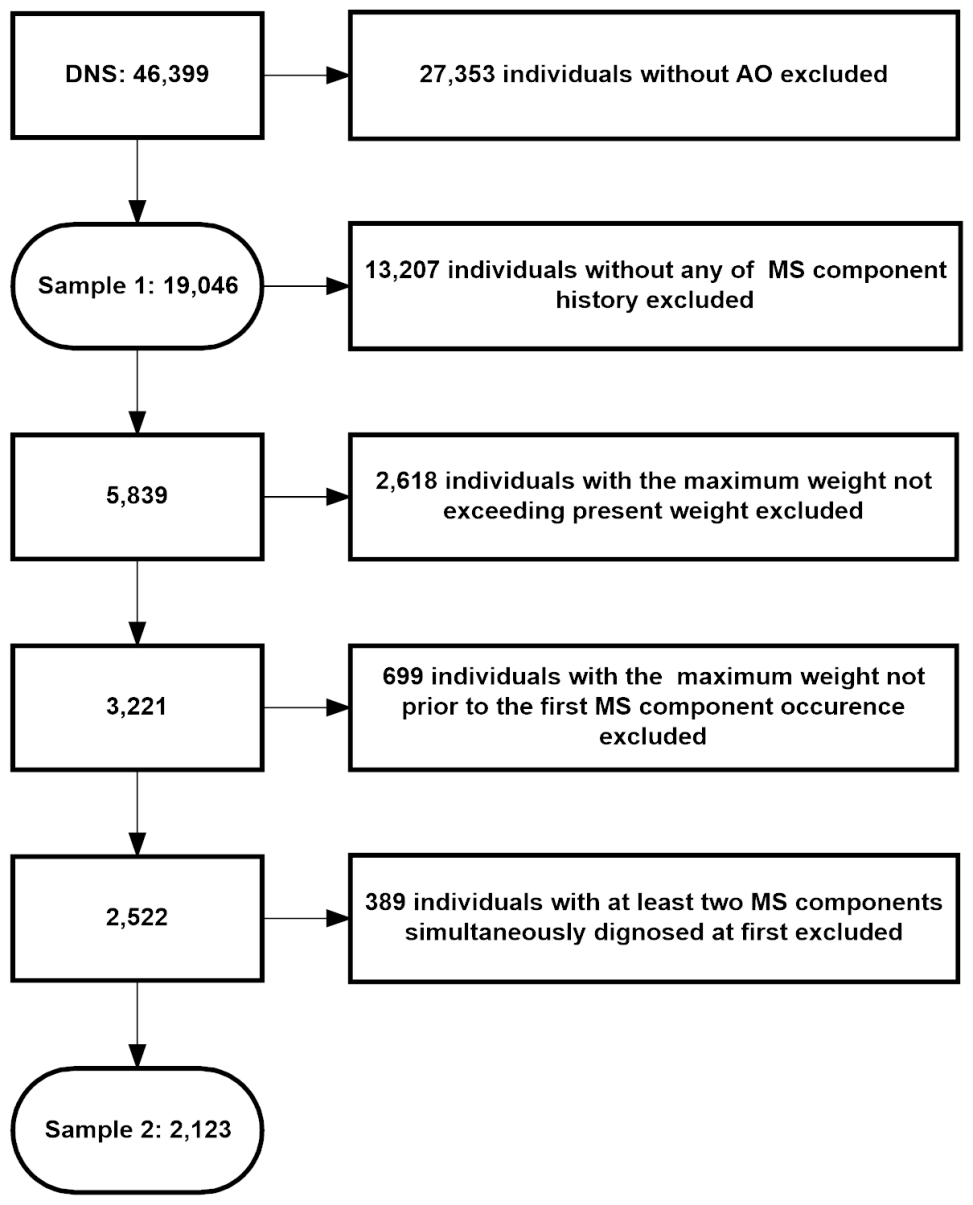
Flow chart of study participants. Abbreviations: MS: metabolic syndrome; AO: abdominal obesity.

Among 46,399 participants in the survey, 27,353 participants without AO were excluded, while 19,046 participants with AO were selected as Sample 1 to study the prevalence of MS and its components. To investigate the process from the first appearance of an abnormal metabolic component to the development of MS, we further chose participants who met the following conditions: 1) AO; 2) a diagnosis history of at least one abnormal metabolic component, so that the first abnormal metabolic component had occurred. We used total cholesterol instead of HDL-c as one of the components, because diagnosis history of HDL-c was not available. People with TC ≤ 6.08 mmol/l or receiving cholesterol-lowering treatment were selected [32]. The feasibility was analyzed in detail, as described in the Discussion section; 3) To ensure that subjects had been in a state of obesity, previous maximum weight was required to be greater than the current weight; 4) To ensure that obesity appeared before the occurrence of the first abnormal metabolic component, the age at which maximum weight was reached was to be prior than the age when the first component happened; and 5) Two or more components could not first have been diagnosed at the same time. Otherwise, the MS may already have developed, and analyses could not be pursued. After the above selection, only 2,123 participants were chosen as Sample 2 for the study. (Figure **1**)

### Data Analysis

Data were analyzed using SPSS 18.0 or Stata 11.0 software for Windows. Data were expressed as mean ± SD, median with interquartile range, or percentage as suitable. Comparisons between groups were analyzed by *t*-test or Mann-Whiteney *U*-test for measurement data, and chi-square test for enumeration data *P* < 0.05 was defined as the threshold for statistical significance. 

The prevalence of MS and its components in Sample 1 was analyzed and gender differences were compared. Age-standardized point prevalence estimates and confidence intervals (95% CI) for MS and its components stratified by sex were calculated using Stata (version 11.0) *svy* commands to account for the multi-stage stratified random sampling design. The calculation was weighted on the basis of Chinese population data from 2006 [33]. The presence of two or more of four components of MS could be diagnosed as MS. Thus, people with AO have a total of 10 combinations of components, including six kinds of combinations of two components, three kinds of combinations of three components, and one kind of combination of four components. The distribution characteristics of each component at different ages, and combinations of different components were analyzed by gender.

Survival analysis was used to study participants with AO in Sample 2 from the occurrence of the first abnormal component to the development of MS. The initial event was defined as the first occurrence of an abnormal component, while the end event was defined as the development of MS. The study time was defined as the time period between the initial event and the end event. If MS had not yet developed, it was defined as a censored value. Gender differences in prevalence were compared between participants aged < 50 years and those aged ≥ 50 years. A survival curve was plotted using Kaplan-Meier method and cumulative prevalence was calculated by life table method; gender differences were analyzed by Cox regression analysis. Andersen 95% confidence intervals for the median survival time of groups were constructed by a forward stepwise method.

## Results

### Characteristics of study subjects

In Samples 1 and 2, the age and maximum weight age of males were significantly lower than those of females (*p* < 0.05). There was significant gender difference in educational level and lifestyle risk factors as cigarette smoking and alcohol drinking. The height, current and maximum weight, body mass index, waist circumference, diastolic blood pressure and triglyceride in males were significantly higher than those in females (*p* < 0.05), while the HDL-c in females was significantly higher than that in males (*p* < 0.05). There was no gender difference in TC in Sample 1 or in fasting blood glucose or systolic blood pressure in Sample 2 (*p* > 0.05). However, TC was significantly higher in females than that in males in Sample 2 (*p* < 0.05). (Table **1**)

**Table 1:** Characteristics of sample 1 and 2 according to gender.

**Variable**	**Sample 1**			**Sample 2**	
	**Male**	**Female**		**Male**	**Female**
n	6,261	12,785		742	1,381
Age, years	47.6 ± 13.4	49.9 ± 12.4*		52.5 ± 12.0	56.5 ± 10.1*
Ethnics (Han), n (%)	5,667 (91.1)	11,624 (91.5)		686 (93.0)	1295 (94.0)
Educational level, n (%)					
College or above	1,647 (26.5)	1,455 (11.4)*		170 (23.1)	116 (8.4)*
Secondary school	3,511 (56.4)	6,667 (52.4)*		427 (58.1)	694 (50.4)*
Elementary school or below	1,066 (17.1)	4,598 (36.2)*		138 (18.8)	566 (41.1)*
Urban, n (%)	2050 (32.7)	4,549 (35.6)*		210 (28.3)	426 (30.8)
Cigarette smoking, n (%)	2,945 (47.1)	431 (3.4)*		348 (47.0)	58 (4.2)*
Alcohol drinking, n (%)	3,041 (48.8)	544 (4.3)*		376 (50.8)	52 (3.8)*
Physical activity, n (%)	2,346 (37.7)	4,696 (36.9)		350 (47.6)	657 (47.8)
Height, cm	169.6 ± 6.5	157.4 ± 6.0*		169.2 ± 6.0	156.5 ± 6.0*
Current weight, kg	79.5 ± 10.1	65.0 ± 8.8*		79.2 ± 9.7	65.0 ± 8.8*
Body mass index, kg/m^2^	27.6 ± 3.1	26.2 ± 3.3*		27.7 ± 3.0	26.5 ± 3.2*
Maximum weight, kg	82.4 ± 11.2	67.9 ± 9.5*		84.2 ± 10.6	69.4 ± 9.1*
	(*n* = 5,943)	(*n* = 11,951)			
Age at maximum weight, years	41.1 ± 12.4	42.0 ± 12.5*		41.6 ± 10.5	42.9 ± 11.3*
	(*n* = 5,261)	(*n* = 10,428)			
Average waist, cm	96.6 ± 6.2	87.9 ± 6.9*		96.7 ± 5.9	89.0 ± 7.1*
Fasting plasma glucose, mmol/l	5.7 ± 1.6	5.6 ± 1.6*		6.4 ± 2.2	6.3 ± 2.3
Systolic blood pressure, mmHg	131.8 ± 18.6	127.8 ± 20.1*		139.3 ± 19.4	138.4 ± 20.1
Diastolic blood pressure, mmHg	84.9 ± 11.5	80.2 ± 11.1*		88.4 ± 12.4	84.2 ± 11.5*
Fasting triglycerides, mmol/l	2.1 ± 1.4	1.7 ± 1.1*		2.2 ± 1.4	1.9 ± 1.2*
HDL-cholesterol , mmol/l	1.2 ± 0.3	1.3 ± 0.3*		1.2 ± 0.3	1.3 ± 0.3*
Total cholesterol, mmol/l	5.0 ± 1.0	4.9 ± 1.0		5.0 ± 1.0	5.2 ± 1.0*

Abbreviations: HDL: high-density lipoprotein.

Data were expressed as mean ± SD, median with interquartile range, or percentage as suitable.

* Male *vs.* Female *p* < 0.05.

### Gender difference in the prevalence of individual components and MS

The age-standardized prevalence of MS in people with AO was 50.5%. The prevalence in males (73.7%) was significantly higher than that in females (36.9%). Hypertension as one of the MS component had the highest age-standardized prevalence (48.9%), followed by hypertriglyceridemia (43.2%), HDL-c (34.0%), and hyperglycemia (32.4%). In males, HDL-c was the component with the highest prevalence (67.7%), followed by hypertension (61.3%) and hypertriglyceridemia (55.7%) showing moderate prevalence, and hyperglycemia having the lowest prevalence (36.4%). In females, the MS component of hypertension had the highest prevalence (41.3%), while HDL-c had the lowest prevalence (14.8%). (Table **2**)

**Table 2:** Age-standardized prevalence of MS and its components in male, female and total participants with abdominal obesity.

**Age duration (years)**	**MS**			**BG**			**BP**			**TG**			**HDL-c**			***p* value**
	**n / N**	**%**		**n / N**	**%**		**n / N**	**%**		**n / N**	**%**		**n / N**	**%**		
Male participants																<0.001
20-30	383/613	62.5		144/613	23.5		293/613	47.8		308/613	50.2		418/613	68.2		
30-40	966/1,296	74.5		424/1,296	32.7		729/1,296	56.3		791/1,296	61.0		939/1,296	72.5		
40-50	1,257/1,613	77.9		649/1,613	40.2		1,042/1,613	64.6		1,019/1,613	63.2		1,041/1,613	64.5		
50-60	1,151/1,473	78.1		664/1,473	45.1		1,048/1,473	71.1		780/1,473	53.0		957/1,473	65.0		
> 60	1,030/1,266	81.4		665/1,266	52.5		1,022/1,266	80.7		594/1,266	46.9		811/1,266	64.1		
Crude	4,787/6,261	76.5		2,546/6,261	40.7		4,134/6,261	66.0		3,492/6,261	54.9		4,166/6,261	66.5		
Standardized*		73.7			36.4			61.3			55.7			67.7		
Female participants																<0.001
20-30	187/849	22.0		177/849	20.8		162/849	19.1		226/849	26.6		146/849	17.2		
30-40	532/1,893	28.1		478/1,893	25.3		533/1,893	28.2		593/1,893	31.1		297/1,893	15.7		
40-50	1,249/3,315	37.7		1,030/3,315	31.1		1,538/3,315	46.4		1,168/3,315	35.2		464/3,315	14.0		
50-60	1,971/3,811	51.7		1,449/3,811	38.0		2,379/3,811	62.4		1,726/3,811	45.3		457/3,811	12.0		
> 60	1,823/2,917	62.5		1,349/2,917	46.2		2,208/2,917	75.7		1,431/2,917	49.1		366/2,917	12.5		
Crude	5,762/12,785	45.1		4,483/12,785	35.1		6,820/12,785	53.3		5,144/12,785	40.2		1,730/12,785	13.5		
Standardized*		36.9			30.2			41.3			35.4			14.8		
Total participants																<0.001
20-30	570/1,462	39.0		321/1,462	22.0		455/1,462	31.1		534/1,462	36.5		564/1,462	38.6		
30-40	1,498/3,189	47.0		902/3,189	28.3		1,262/3,189	39.6		1,384/3,189	43.4		1,236/3,189	38.8		
40-50	2,506/4,928	50.9		1,679/4,928	34.1		2,580/4,928	52.4		2,187/4,928	44.4		1,505/4,928	30.5		
50-60	3,122/5,284	59.1		2,113/5,284	40.0		3,427/5,284	64.9		2,506/5,284	47.4		1,414/5,284	26.8		
> 60	2,853/4,183	68.2		2,014/4,183	48.1		3,230/4,183	77.2		2,025/4,183	48.4		1,177/4,183	28.1		
Crude	10,549/19,046	52.0		7,029/19,046	36.9		10,954/19,046	57.7		8,636/19,046	45.3		5,896/19,046	32.6		
Standardized*		50.5			32.4			48.9			43.2			34.0		

Abbreviations: MS: metabolic syndrome; BG: raised plasma glucose; BP: raised blood pressure; TG: raised triglycerides; HDL-c reduced high-density lipoprotein cholesterol.

Data are expressed as number of cases (% of total).

* The calculations were weighted on the basis of Chinese population data from 2006.

### Gender difference in the prevalence of MS component combinations

**Figure 2:**
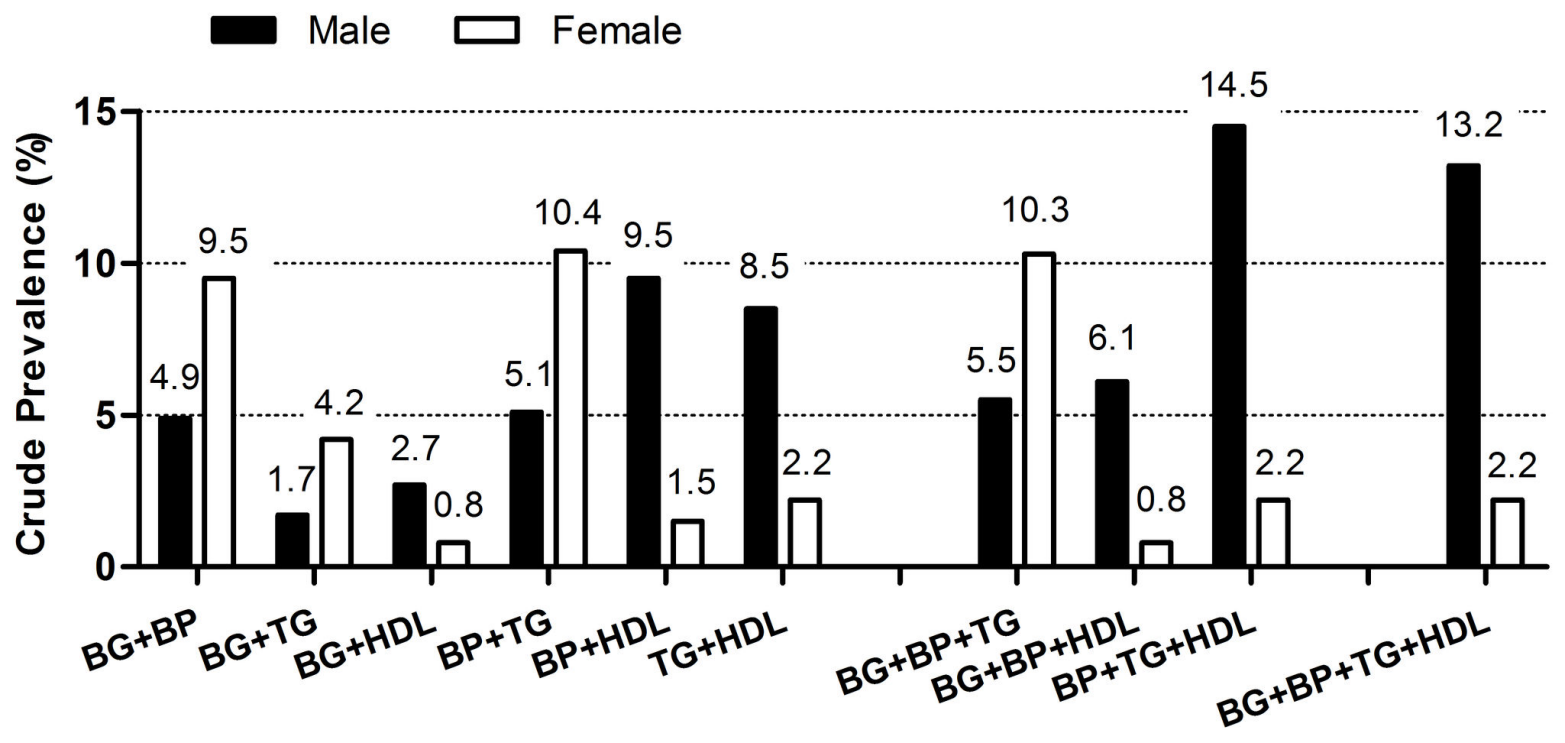
Prevalence of metabolic syndrome components combination in male and female participants with abdominal obesity. Abbreviations: BG: raised fasting plasma glucose, BP: raised blood pressure, TG: raised triglycerides, HDL: reduced high-density lipoprotein cholesterol.

Males had the highest prevalence of MS with the combination of hypertension plus hypertriglyceridemia plus HDL-c components (14.5%), while females had the highest prevalence with the combination of hypertension plus hypertriglyceridemia (10.4%) and hyperglycemia plus hypertension plus hypertriglyceridemia (10.3%). In addition, the proportions of combinations of three and four components were significantly higher in males than those in females (36.4% *vs.* 30.2% and 18.4% *vs.* 5%, respectively). (Figure **2**)

### Gender difference in the development from the initial component to MS

**Figure 3:**
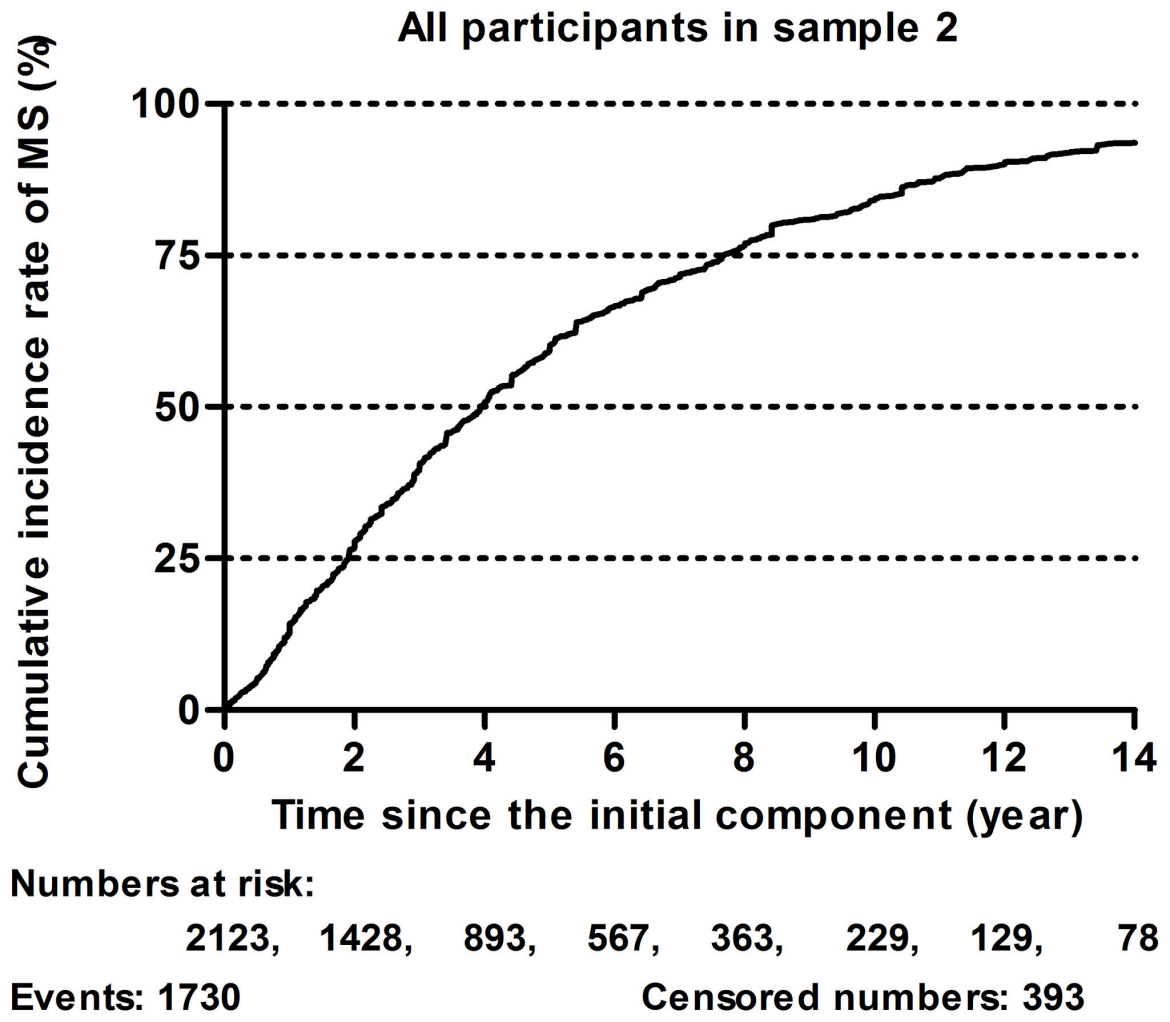
Kaplan-Meier curves of the cumulative incidence rates of metabolic syndrome for all participants in sample 2. Abbreviations: MS: metabolic syndrome.

**Figure 4:**
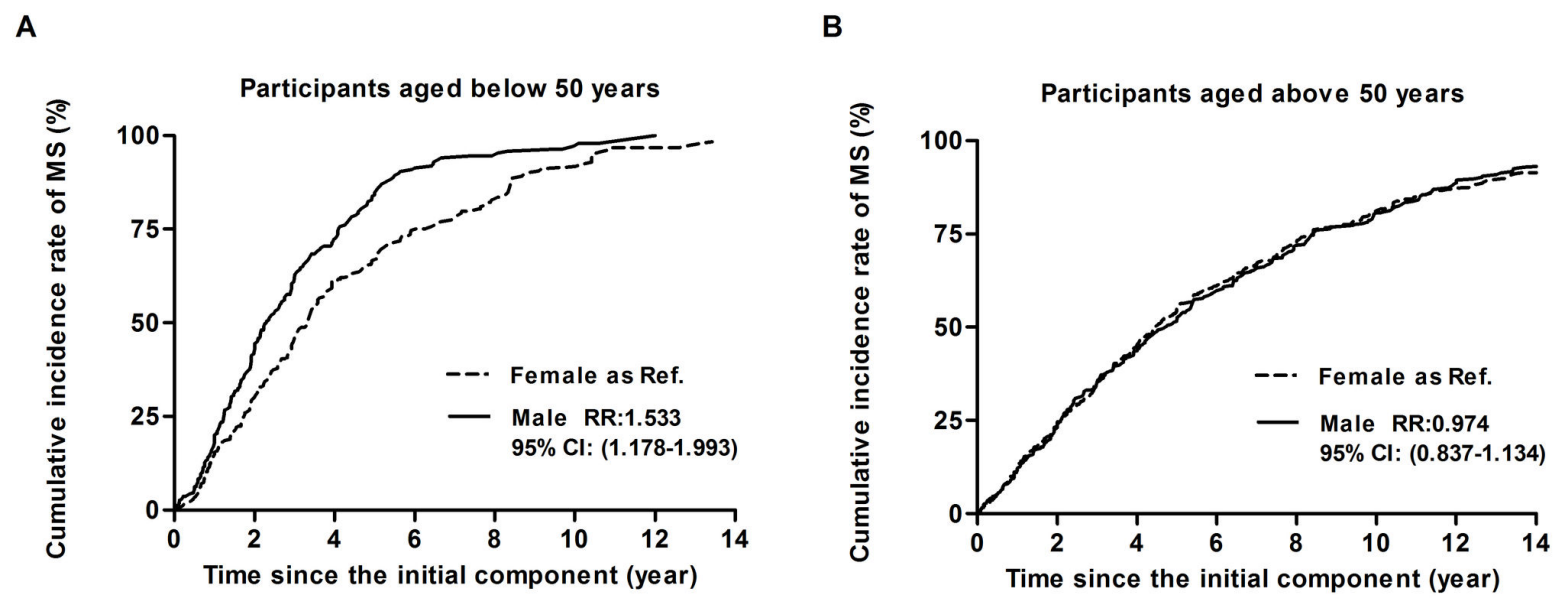
Kaplan-Meier curve of effect of gender for participants in sample 2with ages below 50 years (A) and above 50 years (B) divided by gender. Abbreviations: MS: metabolic syndrome. Male as reference. The relative ratio (RR) and 95% confidence intervals (CI) were calculated by cox regression after adjustment for ethnics, educational level, cigarette smoking, alcohol drinking, and physical activity.

There were 1,730 individuals that developed MS among the 2,123 participants with AO in Sample 2, within the total observation time. MS developed fast at first and then became slow down later. The 5-year and 10-year cumulative prevalence of MS were 58% and 83%, respectively. Half of the participants with AO developed MS after 3.9 years (95% CI: 3.7 - 4.1), whereas 75% developed MS after 7.7 years (95% CI: 7.5 - 7.9) (Figure 3). After adjusted for ethnics, educational level, cigarette smoking, alcohol drinking, and physical activity, males developed MS significantly faster than females in the group aged < 50 years (RR: 1.533, 95% CI: 1.178–1.993), whereas there was no significant difference in the development of MS between males and females aged ≥ 50 years (RR: 0.974, 95% CI: 0.837–1.134) (Figure **4**).

## Discussion

The present study focused on the prevalence and characteristics of MS in a Chinese population with AO, which, as far as we have known, have not been reported previously in China. Our results were obtained using data from a whole population-based national sampling survey that is more representative than previous hospital- or community-based studies on MS with AO. In addition, we analyzed the process of MS from the occurrence of the first abnormal MS component to the development of MS, which has been scarcely reported in the past.

Previous studies have shown that the prevalence of MS in general Chinese population was about 5–30%, depending on the diagnostic criteria used [16,18-21]. In Table 2 of our study, the age-standardized prevalence of MS in participants with AO was 50.5%, which was significantly higher than that in general population. The prevalence of MS in males (73.7%) was almost two times that in females (36.9%). This gender difference was different from that in general population. For example, Li et al. reported the age-standardized prevalence of MS in males of the general Chinese population was only 1.5-fold that in females (15.7% vs. 10.2%) [17]. There are even two studies showing that, in the general population, the age-standardized prevalence of MS in males is significantly lower than that in females [19,34]. In addition, consistent with Claudio L. Lafortuna’s study [10], we found the MS component of hypertension had the highest age-standardized prevalence in all study subjects with AO (48.9%). However, only in females did hypertension have the highest prevalence (41.3%); the component with the highest prevalence in males was HDL-c (67.7%). The gender difference in the prevalence of different components in AO population is significantly different from previous general Chinese population. For example, InterASIA study based on the national whole Chinese population sampling survey showed that hypertension was the most frequent component among males (44.2%), whereas HDL-c was the most frequent component in females (46.5%) in the general population [34]. There are some reasons that account for the difference between ours and InterASIA study. The first is the age difference of participants between the two studies. We included adults aged above 20 years while the age range in InterASIA Study was 35-74 years. The second is the parameter of HDL cholesterol in MS criteria. The cutoff in our study was 1.0 mmol/L in men or 1.3 mmol/L in women, while it was 1.0 mmol/L for both men and women in InterASIA Study. Even so, we speculate that AO might influence men’s dyslipidemia more than female’s, which needs further studies to elucidate in the future.

Based on the analysis of MS components according to age groups in Table 2, we firstly revealed there were significantly more people with AO having hypertension as they aged. The prevalence of hypertension increased even up to 75% in those with AO aged >60 years, and males showed higher prevalence of hypertension than females in all age groups. The high prevalence of hypertension could be explained by the following reasons: on the one hand, the law of hypertension, namely that blood pressure increases significantly with age, indicates that age has significant effects on blood pressure [35,36]; on the other hand, the diagnostic criteria for blood pressure may be too rigid and lack age-specific diagnostic cut-off points [37,38], because it is well known that the normal value of blood pressure in elderly people are higher than those in younger people [39]. Secondly, we found that the prevalence of the hyperglycemia component in both genders was similar, i.e. it increased gradually from ~20% at 20 - 30 years old to ~50% at age >60 years. Thirdly, one component that differed in frequency between males and females in different age groups was hypertriglyceridemia. The prevalence of hypertriglyceridemia in males at different ages was about 50%, with slight variations among different age groups, while its prevalence in females increased gradually with age. Lastly, we showed the biggest difference between males and females was HDL-c component. The prevalence of the HDL-c component in females with AO in different age groups was relatively low and decreased slowly with age, but it was high in males with AO, remaining at 60 - 80%. This significant gender difference in MS components in different age groups is of great importance for differential prevention strategies in different age groups.

The combinations of different MS components were compared between males and females in Figure 2. Consistent with the finding that the prevalence of MS in males was higher than that in females, the combination of MS components was more complicated in males than in females. Besides AO, there were higher prevalence of two, three and four components combinations in males than in females. Furthermore, the proportions of three and four combinations in males were significantly higher than those in females (40.6% vs. 30.1% and 9.0% vs. 5.0%, respectively). It is a commonly held view that combinations of more components equates to a higher risk of CVD [24]. In addition, a prospective study at the Framingham Heart Center showed only two kinds of combinations were associated with CVD and mortality, i.e. hyperglycemia plus hypertension (plus AO) and HDL-c plus hypertension plus hypertriglyceridemia [40]. We found that males showed higher prevalence than females for the above two combinations among people with AO (29.7% vs. 22.8 and 27.7% vs.4.4%, respectively; data not shown). Thus, we concluded that males with AO showed more complex and risky combinations of MS compared with females.

Finally, we analyzed how people with AO developed MS from the occurrence of the first abnormal component in Figure 3 and 4. It was found that MS developed quickly, and then its development slowed down. Half of participants with AO developed MS within 5 years, which is somewhat faster than that described in Hwang’s report [23]. We found at the same time that 75% of participants with AO developed MS within 10 years. Our results suggest that the first 5 years after the occurrence of the first component is critical and should be paid more attention. Further, this attention ought to be maintained for up to 10 years. In addition, because menopause significantly affects females [41,42], we divided the study subjects into two groups, those aged <50 years and those aged >50 years. We found that the speed of development of MS in males aged <50 years was significantly faster than that in females of the same age (RR: 1.533, 95% CI: 1.178–1.993); whereas there was no statistically significant difference between males and females aged >50 years. Our results in the population with AO are different from the development characteristics of MS in general population. For instance, Hwang et al. reported the prevalence of MS in menopausal women was significantly lower than that in men, but, after menopause, females gradually caught up and passed males in terms of the prevalence of MS. These authors hypothesized that obesity might cause the difference in MS development before and after menopause in women [23]. We showed that there was no gender difference between males and females aged >50 years because we selected only participants with AO. Our result could partly confirm their hypothesis.

### Limitations

The main limitation of our study is that TC was used as one component instead of HDL-c, because we could not obtain diagnostic history of HDL-c in China. There are two reasons for choosing TC to replace HDL-c. One reason is that the purpose of studying MS was to screen people at risk of CVD and diabetes, while TC has already been demonstrated to be associated with CVD [43-46]. The other reason is that it was still a feasible method, although we might screen different populations using TC as an alternative component. Previous studies have shown that about 21% of the high-risk population was filtered out as MS using three official criteria. However, only 9% of the high-risk population was screened by all three standards; in other words, about half of the screened population was different for each standard [3]. In fact, although not intending to compare, we discovered that multiple metabolic indexes (e.g. waist circumference and blood pressure) were higher in Sample 2 than in Sample 1 in the present study, which indicated that we might have overestimated the rate of development of MS to some extent. However, due to a lack of prospective studies, the use of TC as an alternative component is reasonable and feasible for studying the characteristics of MS development. 

The second limitation of the study is recall bias, including previous weight and diagnostic history of abnormal components, which were unavoidable for a retrospective study. Another concern is whether people with abnormal components will be diagnosed in time. Thus, we randomly selected some of our subjects and called them back to ask about their previous history. It was found that the consistency of memory was 87% and the Kappa value was 0.78. Considering that AO people experiencing the occurrence of the first abnormal component will be concerned about their health status and take regular physical examinations, we believe that the above reasons could dispel any concerns about recall bias to some extent.

In addition, the number of females was higher than that of males because females had a relatively higher response rate than males, which might cause a potential selection bias [27].

## Conclusion

In summary, we analyzed gender differences in the prevalence and development of MS and its components in a Chinese population with AO. We found the prevalence of MS in the AO population was significantly higher than that in the general population. Compared with females, males should receive more attention because of their higher prevalence of MS and its components, more complex and risky combinations of abnormal components, and faster development of MS. In addition, we revealed that MS developed fast at first and became slow down later. The first 5 years after the occurrence of the first component in people with AO is very critical and should be paid attention, which ought to be maintained for up to 10 years. Our study is of importance for augmenting the knowledge of MS and its components in the population with AO, which is obviously different from that in general population.

## Supporting Information

Appendix S1 **China National Diabetes and Metabolic Disorders Study Group.**
(DOC)
